# The Influence of Horse Age, High-Pressure Technique and Various Heat Treatment Methods on the Quality of Horse Meat

**DOI:** 10.3390/molecules30183749

**Published:** 2025-09-15

**Authors:** Renata Stanisławczyk, Jagoda Żurek, Mariusz Rudy, Marian Gil, Anna Krajewska, Dariusz Dziki

**Affiliations:** 1 Department of Agricultural Processing and Commodity Science, Institute of Food and Nutrition Technology, Faculty of Technology and Life Sciences, University of Rzeszow, Zelwerowicza 4, 35-601 Rzeszów, Poland; mrudy@ur.edu.pl (M.R.); mgil@ur.edu.pl (M.G.); 2 Department of Finance and Accounting, Faculty of Economics and Finance, University of Rzeszow, Cwiklinskiej 2, 35-601 Rzeszów, Poland; jzurek@ur.edu.pl; 3 Department of Thermal Technology, University of Life Sciences in Lublin, Głęboka 31, 20-612 Lublin, Poland; anna.krajewska@up.lublin.pl

**Keywords:** HHP, sous-vide, physicochemical properties, textural profile, sensory properties, chemicalcomposition

## Abstract

The aim of this study was to demonstrate the effect of horse age, high-pressure cooking, and various heat-treatment methods on the quality of horse meat. The research material consisted of samples of the longissimus thoracis muscle obtained from 14 horse carcasses from two age groups. Samples of the *longissimus thoracis* muscle were subjected to traditional cooking (TC), sous-vide cooking (S-V), high-pressure cooking (HHP), HHP + TC, and HHP + S-V. The chemical composition, physicochemical properties, color parameters, pigment levels, texture parameters, and sensory properties of the meat were determined. Exposing horsemeat samples to high pressureand in combination with various heat treatment methods resulted in a color change, increasing the lightness (L*) and decreasingthe metmyoglobin (Mb•O_2_) level. It was found that the combination of treatments used in both age groups resulted in an increase in texture parameters of horse meat samples compared to the control sample (*p* < 0.05). The use of the HHP, HHP + TC, and HHP + S-V techniques led to a significant increase in the TBARS index in both age groups to a level above 2 mg MDA/kg compared to the control samples. Exposing horse meat to TC and the combination of HHP + TC and HHP + S-V resulted in increased weight loss, which ranged from 42.91% to 48.56%.

## 1. Introduction

Modern preservation methods that can be used in meat production include ultrasound, pulsed electric and magnetic fields, pulsed light beam, low-temperature plasma technology, vacuum packaging combined with shrinking, and high-pressure techniques [1]. In recent years, intensive work and research have been conducted to expand the possibilities of using high-pressure technology in the food industry, including meat processing, both as a non-thermal method of food preservation and its processing, including the creation of products with new functional and sensory properties. In the method using high hydrostatic pressure, abbreviated as HHP (High Hydrostatic Pressure), liquid or solid food products are placed in a pressure chamber where they are uniformly subjected to hydrostatic pressure ranging from 100 to 1000 MPa throughout their entire volume for 5–20 min. The use of high hydrostatic pressure in the meat industry can have practical applications in improving the quality of raw materials and processed products [2,3]. Many authors [4,5,6,7] report that the use of high pressure inactivates microorganisms and enzymes in food. Inactivation of microorganisms in meat is usually achieved at pressures above 400 MPa [2]. The use of high-pressure technology can also have a significant impact on texture profile. High-pressure technology has been identified as a physical process for softening (tenderizing) meat and meat products without the use of additives [8]. As a result of HHP, the nutritional value, vitamin content, and most of the substances responsible for the product’s flavor remain unchanged [9]. The use of pressure above 200 MPa causes a drastic color change in red meat within minutes. This is caused by the oxidation of myoglobin to metmyoglobin [10,11]. Under the influence of high pressure, meat becomes lighter and may take on a gel-like consistency, thus losing the typical appearance of fresh raw material [12]. The use of high pressure alters the properties of muscle proteins. They undergo physicochemical changes, such as denaturation, dissociation, solubilization, aggregation, and gelation, the rate and extent of which depend on the pressure level, temperature, pH, and ionic strength [13,14]. Sarcoplasmic proteins, primarily enzymes and heme pigments, are highly susceptible to denaturation under pressure above 200 MPa, which alters the water-holding capacity and color of the meat [15]. Myofibrillar proteins are associated with the meat structure and decompose when the pressure is 300 MPa or higher [16,17,18,19]. An unfavorable phenomenon occurring in meat subjected to pressure is lipid autoxidation, particularly those with a high proportion of polyunsaturated fatty acids. The main cause of these unfavorable reactions is believed to be the denaturation of heme proteins by pressure and the release of Fe(II) or Cu(II) ions, which accelerate lipid autoxidation in the pressurized meat [20].

Currently, the meat industry uses many methods to eliminate unfavorable meat quality characteristics, such as dark color or unsatisfactory tenderness, especially when obtained immediately after slaughter. Horse meat is an example of such a raw material, especially from older animals. Unlike meat from other animals, horse meat contains a high content of connective tissue (collagen) [21] and a high content of the muscle pigment myoglobin [22,23]. It should be emphasized that age causes significant variation in the obtained raw material. With maturity and increasing age of the animal, not only does the chemical composition of the obtained meat change, but also the mechanical stability of the connective tissue increases due to collagen cross-linking, and the meat color becomes increasingly darker. The often unsatisfactory quality of horse meat is also related to high levels of glycogen, which is responsible for the characteristic sweet taste of this raw material [24].

Despite numerous reports on the use of high-pressure heat treatment in beef [10,25,26,27], pork [28,29,30], turkey [16,31], lamb [32], and fish [20], there are currently no studies demonstrating the effect of high-pressure heat treatment on the quality of horse meat. Despite the use of a wide range of heat treatment parameter combinations and the extensive analysis conducted in previous publications [33,34,35], it has not been clearly established which experimental factors improve all quality parameters of horse meat. Therefore, this publication is a continuation of research aimed at identifying methods or combinations of methods that will comprehensively improve the quality of horse meat, especially from older horses. For this reason, in this study, the high-pressure technique was used and this type of processing was combined with the traditional cooking method in a bag (temperature of 100 °C for 1.5 h) and the sous-vide heat treatment method, which used a higher temperature of 85 °C for 4 h.

Therefore, the aim of this study was to assess the effect of horse age, high-pressure techniques, and various heat-treatment methods on the chemical composition, physicochemical properties, color parameters, heme pigment levels, texture parameters, and sensory properties of horse meat. Obtaining more favorable quality characteristics for meat treated with high pressure alone, or in combination with various heat-treatment methods, would indicate the usefulness of these techniques in improving the quality of horse meat, particularly from older animals. Furthermore, due to the differences in some quality characteristics of meat from carcasses of younger and older horses, it is advisable to conduct research to determine which type of processing will be more effective in improving the quality parameters of horse meat from the two age groups.

## 2. Results and Discussion

### 2.1. Chemical Composition

Table 1 presents data on the chemical composition of horse meat. These data indicate that the interaction effect between age and processing type had a statistically significant effect on the protein content of horse meat (*p* < 0.05). A tendency toward increased protein content was observed with increasing age of the horses, although statistically significant differences were only confirmed for meat samples treated with HHP + TC. The content of the remaining basic chemical components contained in the tested raw material, i.e., fat and water, was statistically significantly influenced by the age of the animals, the type of processing used, and the interaction effect between age and processing type (*p* < 0.05). With increasing age of the horses, a tendency toward significantly reduced water content and significantly increased fat content was observed in all tested horse meat samples exposed to a wide range of combined treatments.

The trend toward increasing protein and fat content and decreasing water content in horse meat with age is confirmed by current scientific literature [36,37,38,39]. The literature does not provide specific and comprehensive data on the effect of HHP on the chemical composition of meat Cooking significantly impacts the chemical composition of meat, particularly its water and protein content, leading to denaturation and increased digestibility. It also changes the meat’s structure and causes weight losses of up to 35% during cooking. This process also affects the content of fats, vitamins, minerals, and extracts, and can lead to the formation of new compounds with different flavor and nutritional profiles. The sous vide method, on the other hand, preserves the meat’s nutritional value thanks to low-temperature cooking in sealed bags, minimizing vitamin and mineral loss. It improves the texture and juiciness of the meat through precise temperature control, preventing it from drying out. Considering the type of processing used, it was shown that the combination of treatments did not significantly affect the protein content but did influence the content of other chemical components present in horse meat, i.e., water and fat. The lowest water content in both age groups was found in horse meat samples treated with HHP + TC. In horse meat from younger animals, the lowest fat content was observed after the application of the HHP technology, while in meat from the second age group, after the combination of HHP + S-V technologies. In previous studies by the authors of the publication [35] on the effect of different types of thermal processing on the quality of foal meat, it was shown that foal meat samples subjected to S-V treatment (55 °C, 65 °C for 4 and 24 h) were characterized by a significantly lower fat content and a significantly higher water content compared to cooking in a traditional way in water at a temperature of 100 °C in a bag for 1.5 h. In the case of protein, the type of thermal processing used did not significantly affect the content of the above-mentioned chemical component in the tested raw material.

### 2.2. Physicochemical Properties

Table 2 presents data on the physicochemical properties of horse meat. Two-factor analysis of variance showed that animal age had a statistically significant effect on the hydration properties of horse meat (*p* < 0.05). Also, the interaction effect between HHP and age did not influence the differentiation of these properties.

The data in Table 2 indicate that horse meat from carcasses of older animals is characterized by a greater ability to retain water within the muscle structure, which is reflected in lower forced and thermal drip loss. This relationship is confirmed by other studies [40]. The use of the high-pressure technique did not significantly affect the hydration properties of horse meat. Vaudagna et al. [41] also demonstrated that neither the pressure level nor the holding time (1 or 5 min) affected the water holding capacity (WHC) of cured beef carpaccio subjected to pressure of 400 or 650 MPa at 20 °C. In the study by Kim et al. [42], the application of high pressures at 100 MPa did not result in a change in the WHC of beef semitendinosus muscle, while an increase in pressure to 200–500 MPa led to a decrease in water retention capacity compared to control samples. This phenomenon can be explained by the fact that high pressure causes the relaxation of intra- and intercellular structures, the creation of new spaces accessible to water, and thus the enlargement of existing ones [43]. Pressure-induced denaturation of sarcoplasmic proteins may, to some extent, also contribute to the loss of water retention capacity in muscle tissue exposed to high pressure.

An unfavorable phenomenon occurring in meat subjected to high pressure is lipid autoxidation, particularly those containing a high proportion of polyunsaturated fatty acids [20]. In this study, the presence of secondary fat oxidation products, which formed colored complexes with thiobarbituric acid, was detected in all horse meat samples (Table 2). Animal age, as well as the interaction between age and processing type, had a statistically significant effect on the TBARS index values in horse meat. Significantly higher TBARS values were obtained for horse meat samples obtained from older animals, indicating that such raw material is more susceptible to oxidation processes.

Considering the type of processing used, it should be noted that subjecting horse meat to the combination of HHP, HHP + TC, and HHP + S-V resulted in a significant increase in the TBARS index in horse meat compared to control samples and samples treated with TC and S-V. It should be noted that the obtained TBARS values exceeded 2 mg MDA/kg, i.e., the critical value of malondialdehyde concentration at which oxidative changes can be detected by consumers [44]. The type of raw material analyzed undoubtedly influenced the obtained distribution of results. Horse meat contains a large amount of unsaturated fatty acids [45,46,47], which makes this raw material more susceptible to oxidation processes occurring during thermal processing. In this study, the lowest TBARS values were obtained for CS. The S-V heat treatment method is a technique where food is cooked at a low, controlled temperature in a vacuum-sealed bag for a long time. Under vacuum and low temperature conditions, oxidation is very limited which undoubtedly influenced the distribution of results obtained in the work. The critical concentration of malondialdehyde (MDA), at which oxidative changes may become noticeable to consumers, has been set at 2 mg MDA kg^−1^. Furthermore, the use of high-pressure techniques combined with heat treatment could have resulted in increased phospholipid hydrolysis, which could have resulted in increased free fatty acid content and an increase in the TBARS index. The applied temperature was supposed to have a synergistic effect on phospholipid hydrolysis. The use of high-pressure techniques combined with high temperature led to changes in the structure of the meat, which could increase the availability of oxygen to lipids, thus promoting oxidation. Additionally, the treatments used may have deactivated the natural enzymatic and non-enzymatic defense systems, thus facilitating oxidation. To stop fat oxidation in meat, you can use natural antioxidants derived from herbs (rosemary, sage, oregano, thyme), vitamin C (ascorbic acid), and fruit extracts (e.g., blueberries), which prevent lipid oxidation processes by capturing free radicals and chelating metals.

The mechanisms of lipid oxidation induced by HHP are not fully understood. It is hypothesized that HHP may accelerate lipid oxidation by increasing iron availability from heme proteins and disrupting intercellular membranes [48]. The main cause of these unfavorable reactions is believed to be denaturation of heme proteins by pressure and the release of Fe (II) or Cu (II) ions, which accelerate lipid autoxidation in the pressurized meat. McArdle et al. [49] demonstrated that in the case of beef, high hydrostatic pressure of 200 MPa had no significant effect on TBARS values. Scientific publications on the effect of HHP on fat oxidation in meat emphasize that pressure of 300 to 600 MPa is crucial for initiating lipid oxidation in fresh pork, beef and poultry, as well as in meat products, which may lead to significant changes in the content of lipids, phospholipids and the composition of fatty acids, including free fatty acids [15,50,51,52]. However, numerous studies indicate that lipid oxidation increases with increasing HHP pressure in chicken [53,54], turkey [31], beef [55] and pork [29,30]. For example, McArdle et al. [49] showed higher TBARS values at 600 MPa compared to samples exposed to pressure up to 400 MPa.

In our earlier work [35], foal meat samples cooked traditionally at 100 °C for 1.5 h showed a TBARS value of 1.04 mg MDA/kg. A lower value of the analyzed parameter of 0.88 mg MDA/kg was achieved by cooking foal meat samples in water to 85 °C at the geometric center of the samples. The same TBARS value was achieved for foal meat samples cooked sous-vide at 65 °C for 4 h. In another study [33] conducted on horse meat treated with S-V, the highest TBARS value of 1.35 mg MDA/kg was shown for samples treated with S-V at 65 °C for 24 h. It has also been shown that the use of higher temperatures and longer durations of S-V heat treatment results in increased lipid oxidation and thus promotes an increase in the TBARS index. Therefore, according to the authors of the cited publication, to minimize TBARS values, it is recommended to perform S-V heat treatment at low temperatures and for a short time.

Analysis of the numerical data regarding water activity values showed that exposing horsemeat to a wide range of combinations, i.e., HHP, TC, S-V, HHP + TC, and HHP + S-V, did not significantly affect the value of the analyzed parameter. Water activity in horsemeat samples ranged from 0.980 to 0.987. Horse age also did not significantly affect the water activity in the analyzed samples of the raw material.

The redox potential (ORP) in meat depends on the concentration of oxidants and reducers present. The more oxidants present in meat, the higher the ORP values obtained, and when the concentration of reducers predominates, the lower the ORP values [56]. The presence of endogenous and exogenous reducers in meat plays a significant role in converting the oxidized form of myoglobin (metmyoglobin) to the reduced form. The numerical data in Table 2 regarding the redox potential indicate that animal age, as well as the interaction effect between age and processing type, had a statistically significant effect on the ORP values in horse meat. Horse meat samples from older animals generally showed higher ORP values. This distribution of results indicates that a higher redox potential is equivalent to a higher content of fat oxidation products (TBARS), which is reflected in this study. Two-way analysis of variance showed that the lowest ORP values, as well as the lowest TBARS values, were observed in both age groups of horses, for horse meat samples treated with TC and S-V, and for CS.

Table 2 presents the numerical data regarding the percentage weight loss of horse meat samples treated with various treatments and by animal age. It should be noted that the type of processing used had a statistically significant effect on the weight loss in horse meat. The lowest weight losses, ranging from 39.28% to 42.00%, were observed for meat samples treated with HHP and S-V, with even lower weight losses ranging from 38.59% to 40.76%. Traditional cooking of horse meat and the combination of HHP + TC and HHP + S-V significantly increased the weight loss of the analyzed raw material compared to samples treated with HHP and S-V. The study conducted on horse meat showed the highest mass loss, at 39.04%, in relation to meat samples cooked traditionally at 100 °C for 1.5 h. The cited authors showed that horse meat samples prepared using the S-V technology were characterized by significantly lower mass loss compared to meat prepared traditionally. The smallest mass loss, approximately 13%, was observed in meat samples subjected to lower temperatures in the sous-vide technology (55 °C) for 4 h [35].

According to Hughes et al. [57], heat treatment of meat increases the stiffness of the myofibrillar structure due to protein denaturation, which in turn is responsible for increased weight loss. Jaelan et al. [27] obtained different results. The cited authors demonstrated that beef and buffalo meat samples treated with HPP had lower (*p* < 0.05) weight loss compared to the control samples. Furthermore, samples treated at 600 MPa had a higher percentage of weight loss compared to meat samples treated at 300 MPa. Other authors’ studies [42,49] also indicate an increased weight loss in meat samples as a result of applying higher pressures ranging from 200 to 400 MPa.

### 2.3. Color Parameters and the Level of Heme Pigments

**Figure 1:**
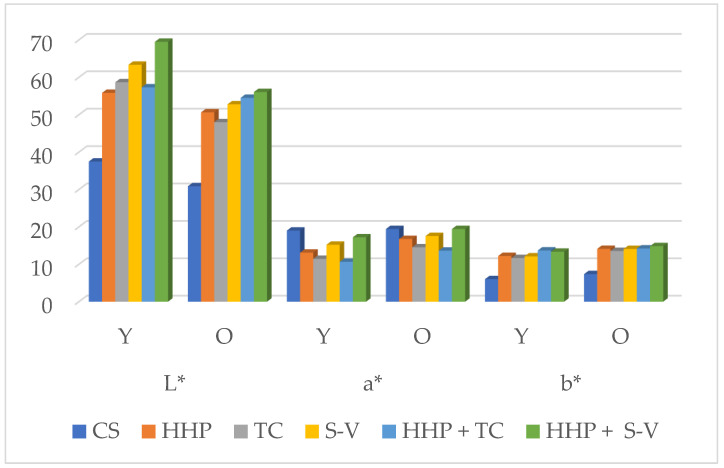
Color parameters of horse meat depending on the processing method; Y—younger horses; O—older horses. Statistical significance of differences between means, ANOVA and *p* values are provided in the tables included in the Supplementary Materials (Table S1).

**Figure 2:**
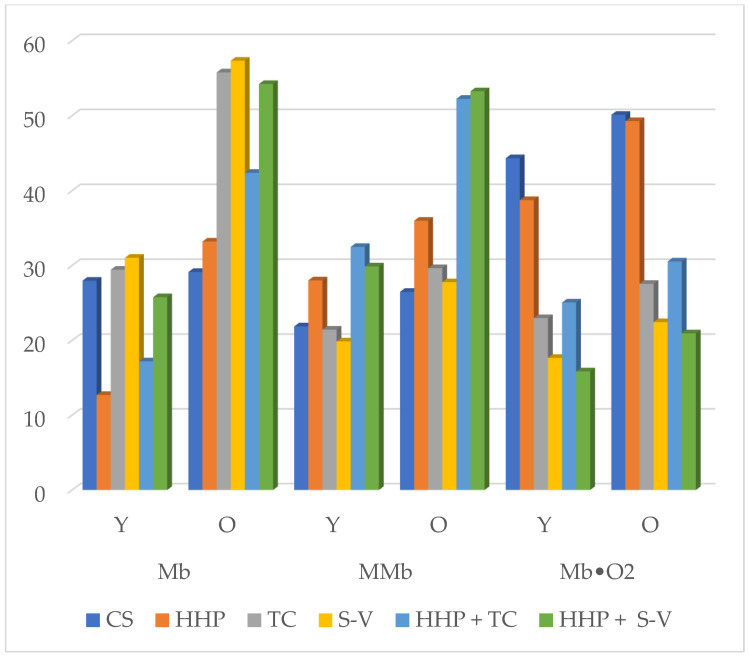
Level of heme pigments of horse meat depending on the processing method; Y—younger horses; O—older horses. Statistical significance of differences between means, ANOVA and *p* values are provided in the tables included in the Supplementary Materials (Table S1).

Studies indicate that the use of high-pressure techniques causes significant changes in the color of fresh meat [2]. Figure 1 and Figure 2 presents data on color parameters and pigment levels in horse meat depending on the age of the animals and the type of processing used. These data indicate that both animal age, the type of processing used, and the interaction between age and processing had a statistically significant effect on the development of selected color parameters and the level of selected pigments in horse meat (*p* < 0.05). Taking into account the age of the horses, significantly higher values of the L* component of horse meat were observed in all meat samples from young animals exposed to a wide range of combinations, except for samples treated with HHP + TC. A reverse trend was observed for the a* color parameter. In all horse meat samples from older animals exposed to various treatment variants, the analyzed a* color parameter values were significantly higher (*p* < 0.05), except for CS compared to meat samples from younger animals.

Many studies [40,58,59,60] emphasize that with age, the color of horse meat becomes increasingly darker, which is due to the high content of the muscle pigment myoglobin [22]. Analyzing Figure 2, it should be noted that horse age had a significant effect (*p* < 0.05) on the pigment levels in horse meat. A significantly higher level of total heme pigments (OZB) was demonstrated in all tested horse meat samples from older animals. Significantly higher levels of myoglobin (Mb) and metmyoglobin (MMb) were also demonstrated in horse meat samples from older animals in all tested horse meat samples, except for the control samples. Taking oxymyoglobin (Mb•O_2_) into account, a significantly higher content of this pigment form was demonstrated only in horse meat samples from carcasses of older animals exposed to HHP.

In this publication, the distribution of numerical values for color parameters and pigment levels in horse meat was also significantly influenced by the type of processing used and the interaction effect between age and processing. Exposing horse meat to a wide range of combined treatments, i.e., HHP, TC, S-V, HHP + TC, and HHP + S-V, resulted in a significant increase in the L* component and the b* color parameter, and a decrease in the a* color parameter, compared to control samples. The obtained results are consistent with those of other authors. Most studies [13,61,62,63,64] found an increase in the lightness component of meat color (L*) within the applied pressure range of 200 to 350 MPa and a change from red to a lighter, pink color.

For example, in a study by Ma et al. [32] conducted on lamb meat, it was shown that the L* values of shank, loin, and shoulder pieces were significantly higher after HHP treatment at 200 MPa, 300 MPa, 400 MPa, and 600 MPa compared to the control samples of the corresponding meat pieces. The red color component (a*) decreased at pressures of 400 to 500 MPa, causing the meat to change to gray-brown with an appearance similar to a cooked product. However, this parameter was more variable and dependent on the type of experiment (i.e., meat type, grinding state, and HHP technology conditions). Under the influence of high pressure, the yellow color value (b*) increased or remained at the same level. The lighter appearance of meat may likely be due to myoglobin denaturation and displacement or release of heme, increased drip losses leading to changes in the water content of the meat, or damage to the porphyrin ring and protein coagulation. Reduction in redness may also be related to an increase in the concentration of metmyoglobin (Fe^3+^) and results in an increased brown coloration of the meat, which is undesirable and responsible for the lack of consumer acceptance of such meat.

Previous studies by the authors of the publication [35] conducted on foal meat showed that cooking horse meat samples in a traditional way, in water at 100 °C for 1.5 h, significantly reduced the color brightness (*p* < 0.05) of this raw material. However, cooking horse meat samples in sous-vide technology (55 °C, 65 °C for 4 and 24 h) resulted in a significant increase in the L* parameter value (*p* < 0.05) compared to traditional cooking. The significantly highest proportion of red color a* was also found in meat samples subjected to S-V heat treatment at 55 °C.

The magnitude and intensity of unfavorable color changes under the influence of HHP depend on the myoglobin content and are more prevalent in fresh red meat, e.g., beef, than in pork, poultry, or cold cuts. Figure 2 regarding pigment levels indicates that significantly the lowest myoglobin (Mb) levels were found in both age groups in meat samples subjected to the high-pressure technique (*p* < 0.05).

The application of TC and S-V to horse meat samples from carcasses of younger animals resulted in a significant increase in Mb levels in the tested raw material. Similarly, subjecting horse meat samples from carcasses of older animals to TC, S-V, and HHP + S-V led to a significant increase in Mb levels (*p* < 0.05). Considering the level of metmyoglobin (MMb), significantly the lowest levels of this pigment form were found in both age groups in horse meat samples subjected to TC, S-V, and CS. In turn, the use of HHP, HHP + TC and HHP + S-V resulted in a significant increase in the MMb level (*p* < 0.05). There is a relationship between fat oxidation and color, as reactive lipid oxidation products enhance metmyoglobin formation. Fat-induced myoglobin oxidation is particularly common in beef. In this study, the highest presence of secondary fat oxidation products was demonstrated in horse meat samples treated with HHP, HHP + TC, and HHP + S-V. These samples showed a significant increase in MMb levels.

Figure 2 indicates that the highest levels of Mb•O_2_ were found in CS and horse meat samples treated with HHP. The remaining treatment combinations resulted in a significant reduction in the level of the analyzed pigment in horse meat. The highest levels of total heme pigments (OZB) were also found in CS. Considering the numerical data presented in Figure 2, it should be confirmed that the age of the horses did not significantly affect the changes in Mb, MMb, and Mb•O_2_ in the control sample group (CS). In this publication, different treatments have a significant impact on the color of the horse meat. Changes in meat color during heat treatment depend on the degree of denaturation of the protein component, myoglobin, which is completely denaturized at temperatures above 70 °C. The HHP method can also cause changes in the color of horse meat. The color changes depend on the myoglobin content and are more visible in fresh red meat than in white meat. The high pressure causes the meat to become lighter and take on a gel-like consistency, losing the typical appearance of fresh meat.

Based on the obtained results, it can be concluded that the combination of treatments used led to a lightening of the color of the horse meat, which may be desirable from the consumer’s perspective. On the other hand, it can be concluded that the most attractive color was observed in the control horse meat samples and those subjected to TC and S-V, due to the lowest percentage of the pigment metmyoglobin, which gives the meat a brownish-gray color, and the highest content of oxymyoglobin, responsible for the meat’s light-red, cherry-red color.

### 2.4. Texture Parameters

Data regarding the texture parameters of horse meat are presented in Table 3. These data indicate that the age of the animals, the type of processing used, and the interaction between age and processing had a statistically significant effect on the shear force required to cut horse meat samples. Considering the age of the horses, it should be noted that in each batch of samples from older animals, the shear force required to cut the meat samples was higher compared to meat samples from younger animals. However, a statistically significantly higher shear force was observed for meat samples treated with HHP + TC. The fact that horse meat, especially from older animals, contains higher amounts of collagen undoubtedly influenced the obtained results. The mechanical stability of connective tissue increases with age due to collagen cross-linking, becoming increasingly compact and less elastic. As animals age, collagen (contained in intermuscular connective tissue) also becomes stiffer, harder, and more resistant to thermal denaturation. This change results from an increase in the number of intermolecular cross-links and their greater stability. Treating horse meat with various treatment options resulted in a significant increase in the shear force required to cut horse meat samples compared to control samples. Previous studies [34] conducted on horse meat subjected to different types of heat treatment showed that subjecting horse meat to the S-V technology (55 and 65 °C for 4 and 24 h) resulted in a significant reduction in the shear force required to cut horse meat samples compared to samples cooked traditionally at 100 °C for 1.5 h. Jaelan et al. [27] conducted a study showing that the shear force required to cut beef and buffalo meat samples was significantly higher when using 600 MPa (82.70 N and 73.38 N) compared to 300 MPa (75.41 N and 66.31 N). In the case of control samples, it was 102.40 N and 85.20 N, respectively. The results of other studies [10,49] also showed the same tendency in the case of beef subjected to pressure of 400 and 600 MPa at a temperature of 20–40 °C.

Hardness 1 is the maximum force recorded during the first compression cycle, while hardness 2 is the maximum force recorded during the second compression cycle. Stiffness up to 5 mm is the force value recorded when the probe is recessed at 5 mm, while stiffness up to 8 mm is the force value recorded when the probe is recessed at 8 mm. Instrumental analysis of texture parameters showed that only the type of processing used had a significant effect on hardness 1, hardness 2, stiffness 5, stiffness 8, and chewiness of horse meat. Treating horse meat with different treatment options resulted in significant increases in the values of the analyzed parameters compared to the control samples. Considering texture parameters, meat samples treated with HHP + TC and HHP + S-V exhibited the most unfavorable properties. Animal age did not significantly affect the levels of the texture parameters listed above. Neither animal age nor the combination of treatments used significantly affected the values of the parameters: cohesion, adhesiveness, resilience, and elasticity of horse meat. HHP causes texture hardening and protein aggregation due to denaturation and the formation of new bonds. The denaturation process involves changing the structure of proteins without degrading them. HHP does not disrupt covalent bonds, but it does alter the structure of proteins. New non-covalent bonds develop, primarily hydrophobic, electrostatic, and hydrogen bonds. Proteins lose their native structure and adopt a new conformation. Aggregation, on the other hand, involves the fusion of proteins into larger aggregates. The resulting larger complexes form spatial networks (e.g., gels), which leads to a hardening of the texture, gelation, and increased product cohesion.

Literature reports that the application of high pressures can cause meat protein softening or hardening, depending on temperature, pressure, and time. These changes are caused by protein denaturation, aggregation, or gelatinization. Differences in results may also be due to differences in the characteristics of meat samples from different animal species. Therefore, it is recommended that processing conditions be carefully controlled to achieve a softening effect on muscle tissue. Ma and Ledward [10] found that beef hardness increases after high-pressure processing of 200 MPa or more at 20 °C. Jaelan et al. [27] showed that meat hardness after HHP treatment tended to increase for beef and decrease for buffalo meat. Furthermore, at 600 MPa, beef had a higher (*p* < 0.05) hardness value compared to the control group, while no changes were observed in buffalo samples (*p* > 0.05). The authors also showed that beef samples treated with HHP had higher chewiness, cohesion, and elasticity (*p* < 0.05), whereas in buffalo meat, the changes in these texture parameters after HHP treatment were not significant (*p* > 0.05). In the study by Ma et al. [32], chewiness increased after HHP treatment, and the increase in this parameter was greater at pressures higher than 200 MPa. Also Cheftel and Culioli [65] and Torres and Velazquez [66] found that meat chewiness increased after the application of the high-pressure technique, but the HPP technique had little effect on the strength of the connective tissue, and the chewiness values were proportional to the strength of the internal bonds.

Hardness, elasticity, cohesion, and chewiness are textural parameters that reflect the degree of meat softness, the ability to resist external force recovery, the tightness of muscle tissue bonding, and bite strength [66]. The effect of HHP on textural properties can be explained by the denaturation of myofibrillar proteins and gel formation [67]. In this paper, the use of the high-pressure technique alone, as well as the combination of HHP + TC and HHP + S-V, resulted in a significant (*p* < 0.05) increase in meat hardness and increased stiffness and chewiness. This phenomenon can be justified by the fact that the use of the high-pressure technique at 400 MPa at 20 °C for 15 min is too low, and the duration of the treatment itself is too short, to achieve a tenderizing effect in horse meat. Horse meat is a raw material containing large amounts of connective tissue (collagen), and in the case of meat in a post-rigor state, no tenderizing effect was observed when applying high pressure of 150 MPa at a temperature of 30 °C even for several hours. When assessing the effect of high pressure on collagen solubility in cooked meat, it was found that pressures of 100 and 200 MPa did not change its thermal resistance, while pressure of 300 MPa applied for 5 and 10 min increased the total amount of soluble collagen. Exposure of raw smoked tenderloin samples to 500 MPa for 30 min at 40 °C resulted in a decrease in the content of soluble proteins (sarcoplasmic and myofibrillar) compared to control samples, indicating partial denaturation of muscle proteins during pressure treatment [68]. Future research should aim to improve the tenderness of horse meat. Proteolytic enzymes (papain, bromelain) can be used for this purpose, or high-pressure techniques can be combined with moderate heating, which can contribute to better textural results. Functional additives (e.g., phosphates in the meat industry, salts) can also be used. High pressure weakens hydrophobic interactions and strengthens hydrogen bonds. Low pressures (<100 MPa) do not affect covalent bonds and, therefore, primary structure. At higher pressures, hydrogen bonds may break, affecting protein secondary structures. Pressures above 150 MPa may affect quaternary structures, and pressures above 200 MPa cause changes at the tertiary structure level. In the pressure range of 100–200 MPa, protein denaturation, aggregation, and transition to a gel phase are observed. The quaternary structure of proteins is most susceptible to changes, and these changes can be reversible or irreversible. Polypeptides often degrade at pressures below 150 MPa, accompanied by a volume reduction of up to 500 mL/mol. At pressures above 200 MPa, changes in tertiary structure may be reversible, while changes in secondary structure may be irreversible. The gelling ability of proteins depends on the level of pressure applied and the duration of its exposure. Protein gelation is the result of thermal denaturation of proteins, which leads to intermolecular covalent and noncovalent interactions, including the formation of disulfide bonds and hydrophobic interactions. Until now, this process was considered solely the result of elevated temperature. However, it has been shown that high pressure causes protein gelation even at room temperature. Furthermore, the beneficial effect of applying high pressure prior to heat treatment on the gelling properties of muscle proteins has been demonstrated. It was found that 10 min heating (70 °C) of sheep muscle homogenates in a low ionic strength solution after pressure treatment (10 min at 150 MPa and 0 °C) enhanced their thermal gelation. However, the combined application of high pressure and temperatures above 40 °C impaired the gelling ability of muscle proteins [20].As a result of the conducted research, technological processes, i.e., HHP and TC, resulted in the deterioration of the tenderness of horse meat. This phenomenon was most likely caused by the denaturation of microfibrillar proteins. High-pressure heat (above 300 MPa) denatures myosin and actin-the basic contractile proteins of meat. During heat treatment (50–70 °C), these same proteins also undergo thermal denature and contraction. The combination of both techniques leads to the intensification of the denaturation process, the aggregation of proteins into ordered gel structures and the consolidation of the stiffened muscle structure. The HHP process itself does not degrade collagen (it does not reach the 60–70 °C temperature required for its degradation into gelatin). When heat treatment occurs after HHP, as was the case in this study, the shortened collagen fibers contract strongly rather than undergo hydrolysis. This resulted in increased connective tissue stiffness and decreased tenderness of the horsemeat. Particularly unfavorable effects are observed at HHP, above 400 MPa and heat treatment above 60 °C. To limit the deterioration of tenderness resulting from the above techniques, i.e., HHP + TC, lower pressure (100–250 MPa) and shorter treatment time (1–3 min) should be used. Furthermore, performing the treatment at a lower temperature (0–20 °C) may result in inhibition of myofibrillar protein aggregation. The most significant effect of pressure was detected in meat for sarcoplasmic and myofibrillar proteins. Sarcoplasmic proteins, mainly enzymes and heme pigments, are highly susceptible to denaturation under pressure above 200 MPa, which changes the water-holding capacity and color of the meat [15]. Myofibrillar proteins are inherent to the structure of meat and degrade at pressures of 300 MPa or more. This results in denaturation, agglomeration, and gel formation [16,17,18,19].

### 2.5. Sensory Properties

**Figure 3:**
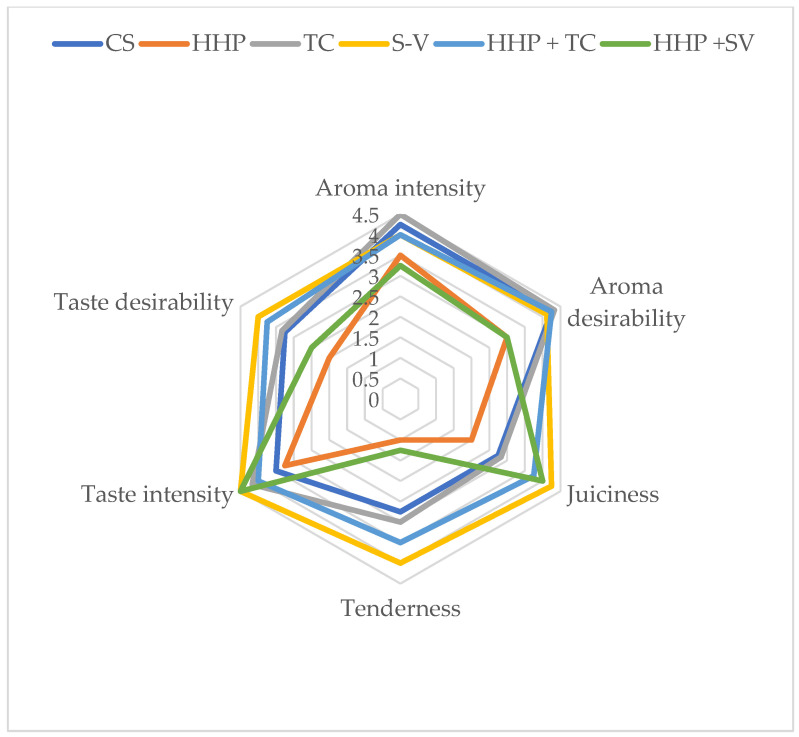
Sensory characteristics of young horse meat depending on the type of processing. Statistical significance of differences between means, ANOVA and *p* values are provided in the tables included in the Supplementary Materials (Table S2).

**Figure 4:**
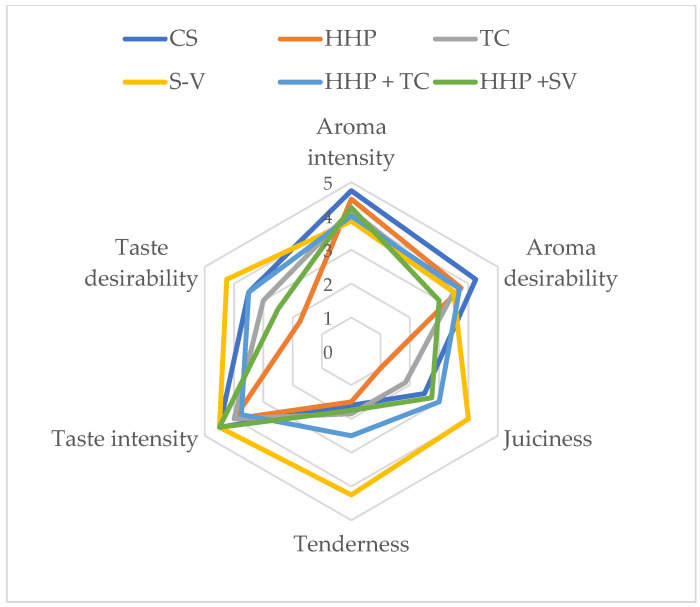
Sensory characteristics of old horse meat depending on the type of processing. Statistical significance of differences between means, ANOVA and *p* values are provided in the tables included in the Supplementary Materials (Table S2).

Unlike heat treatment, which affects both covalent and non-covalent bonds, high-pressure processing conducted at room and moderate temperatures disrupts only relatively weak chemical bonds (hydrogen, hydrophobic, and ionic bonds). High-pressure processing does not alter small-molecule compounds such as vitamins, amino acids, monosaccharides, and flavor compounds. Data on the evaluation of sensory quality parameters of horse meat subjected to various treatments are presented in Figure 3 and Figure 4. These data indicate that animal age did not significantly affect the sensory evaluation parameters of horse meat. The type of processing significantly affected the juiciness, tenderness, and flavor desirability of horse meat.

A significant increase in juiciness was demonstrated in horse meat from younger animals after the application of S-V, HHP + TC, and HHP + S-V (*p* < 0.05). In contrast, in meat from older animals, juiciness was rated highest after the application of the S-V technology. A similar relationship was demonstrated for tenderness and taste desirability. The use of S-V and HHP + TC treatments resulted in the meat from younger animals obtaining the highest scores for the analyzed sensory quality parameters. Considering the tenderness and taste desirability of horse meat from older animals, it must be admitted that the highest scores were awarded to the meat samples treated with the S-V technology. The use of the S-V thermal processing method resulted in improved juiciness, tenderness, and flavor of horse meat in both age groups. Therefore, this type of thermal processing should be considered by the meat industry as a method for improving the specified sensory quality parameters of horse meat. The use of high-pressure cooking alone resulted in the lowest scores for the specified sensory quality parameters. Studies by other authors [35] conducted on horse meat indicate that sous-vide cooking (55 and 65 °C for 4 and 24 h) compared to traditional cooking (100 °C for 1.5 h) significantly reduces the aroma (intensity and desirability) of horse meat. In turn, sous-vide cooking, compared to traditional cooking, significantly increases the juiciness, tenderness, and flavor (desirability) of horse meat.

High-pressure processing is a technology that can affect the sensory properties and consumer acceptance of meat [32]. Morton et al. [69] reported that exposing beef (*m. longissimus thoracis*) to a pressure of 175 MPa for 3 min did not affect the juiciness and flavor of the meat but significantly improved overall acceptability and flavor. After HHP treatment, sample portions were cooled to −1 °C and kept refrigerated for 1 day. The sample batch was then frozen at −20 °C. A set of 25 mm thick steaks was removed from each frozen sample batch for sensory evaluation. The steaks were vacuum-packed, randomly marked, and stored at −40 °C.

Other authors, Rodríguez-Calleja et al. [70], found that chicken breast filet exposed to a pressure of 300 MPa for 5 min was more acceptable and had a greater aroma compared to the control sample. Contrary results were obtained by Kruk et al. [71], who subjected poultry meat to the HHP technique showed that high pressure negatively affected the sensory properties of chicken breast filets. Applying pressure of 300 MPa significantly changed the flavor, aroma, and juiciness of the filets, whereas at pressure of 450 MPa, the meat lost its aroma. Meat samples were subjected to HHP treatment for 5 min at an initial pressure vessel temperature of 15 ± 3 °C. Samples intended for sensory evaluation were stored at 4 °C until use.

## 3. Materials and Methods

### 3.1. Experimental Design

The research material consisted of samples of the longissimus thoracis muscle (*m. longissimus thoracis*) obtained from 14 horse carcasses sourced from individual farmers in southeastern Poland. The horses were divided into two age groups:

I. Younger horses—2 to 7 years old.
II. Older horses—7 to 12 years old.

The age of the horses in this study was determined based on purchase documentation. The pre-slaughter weight of the animals ranged from 450 to 550 kg (500 ± 30 kg). The horses used in the study were of the Malopolski and Silesian breeds. Of the randomly selected horses, 50% were mares and 50% geldings. The selection of carcasses for the study was based on purchase documentation. Participants had no contact with live animals. Before slaughter, which was conducted in accordance with the methodology used in the meat industry and in compliance with European regulations [72], the horses were kept in separate pens in livestock warehouses for approximately 24 h after transport. The animals were stunned using a pin gun before slaughter. To demonstrate the effect of horse age, high-pressure techniques, and various heat treatment methods on horse meat quality, meat samples (1200 g each) were taken from the longissimus thoracic muscle at the level of the 13th-14th thoracic vertebrae. The *longest thoracic muscle* was collected from both younger and older horses (14 half-carcasses × 2 age groups × 6 treatments = 168 meat samples). All muscle samples taken were stored refrigerated before use at 4 °C ± 0.5 °C for a period of 10 days.

### 3.2. HHP Meat Processing and Cooking Procedure

After transport, the collected meat samples were stored under refrigerated conditions (4 °C ± 0.5 °C). Each collected meat sample was divided into six sample batches, across the muscle each weighing approximately 300 ± 30 g (14 half-carcasses × 2 age groups × 6 treatments = 168 meat samples). The division of the collected meat samples into individual batches was made starting from the anterior part of the muscle. The first separated batch of horse meat samples constituted the control sample batch (CS), which was not subjected to any processing. The remaining five batches of horse meat samples were placed in plastic bags and vacuum-sealed using a vacuum sealer (Inauen, Schwanden, Switzerland). After vacuum sealing, the three sample batches were subjected to high hydrostatic pressure. A pressure of 400 MPa was applied at 20 °C for 15 min. The pressure rise time was 100 s, and the pressure drop time was 2–4 s, with the time required to reach the indicated pressure and the decompression time not included in the pressurization times. The pressurization process was performed at the Institute of High Pressure Physics of the Polish Academy of Sciences in Warsaw. A U 4000/65 pressure apparatus (Unipress, designed and manufactured by the High Pressure Laboratory) was used. This apparatus can operate at a maximum pressure of 600 MPa. The second batch of horse meat samples consisted of horse meat samples subjected to high hydrostatic pressure (HHP). The third batch of horse meat samples was cooked traditionally. For this purpose, the collected batch of samples was cooked in a pot in water at 100 °C in a bag for 1.5 h (TC). After heat treatment, all samples were immediately cooled to 4 °C ± 0.5 °C. The next, fourth batch of horse meat samples was heat treated in a water bath [Hendi, Poland] at 85 °C for 4 h (S-V). After heat treatment, the meat samples were immediately cooled to 4 °C ± 0.5 °C. The fifth batch of horse meat samples after HHP treatment was additionally cooked traditionally (HHP + TC) in a pot in water at 100 °C for 1.5 h. After heat treatment, all samples were immediately cooled to 4 °C ± 0.5 °C. The sixth batch of HHP samples was then heat-treated sous-vide (HHP + S-V). For this purpose, the horse meat samples were placed in a water bath (Hendi, Gądki, Poland) and exposed to a temperature of 85 °C for 4 h. After heat treatment, all samples were immediately cooled to 4 °C ± 0.5 °C.

### 3.3. Analytical Methods

Meat samples were analyzed for water content (PN-ISO, 1442, 2000) [73], protein content (based on nitrogen) (PN-A-04018: 1975/Az3, 2000) [74], and fat content (PN-ISO, 1444, 2000) [75]. Meat color was evaluated based on the L*, a*, b* values in the CIE LAB color system, employing the reflectance method with an NR20XE camera (3nh Technology Co., Ltd., Shenzhen, China), following the methodology outlined by [36]. The percentage of heme pigments in the meat samples was determined as outlined by Krzywicki [76]. The amount of thermal drip was determined using Janicki and Walczak’s method [77]. Forced meat drip was assessed using the method of Grau and Hamm [78]. Lipid oxidation was measured through the TBARS (2-thiobarbituric acid-reactive substances) index, applying the methodology outlined by Pikul et al. [79]. Water activity was determined using a Novasina AG LabMaster—aw neo water activity measuring apparatus (Lachen, Switzerland), following the method described by Stanisławczyk et al. [33]. The oxidation-reduction potential (EH, mV) was estimated employing an ERPt-13-type combination electrode and a waterproof pH/conductometer, specifically the ELMETRON CPC-505 model (Zabrze, Poland), following the method described by Stanisławczyk et al. [33]. The loss was determined using the formula below [33].

$$Weightloss\left(\%\right)=\frac{weight\;of\;raw\;meat-weight\;of\;cooked\;meat}{weight\;of\;raw\;material}∙100$$

The shear force of raw meat was determined using TA texture meter. XT plus (Stable Micro System Ltd., Surrey, UK). Samples of raw meat in the shape of cylinders, cut with a 1.0 cm diameter cork borer (along muscle fibers), were cut with a Warner-Bratzler blade with a triangular notch and the value of the force needed to cut them (N/cm^2^) was recorded. The texture parameters were assessed by employing texture profile analysis, facilitated by a CT3-25 texture analyzer (Brookfield, WI, USA). The Texture Pro CT software (V.1.9 Build 39; Brookfield, WI, USA) was used to measure various parameters. The methodology for assessing the texture parameters of the meat samples was previously described in a prior publication [34]. A panel of 12 trained individuals performed the sensory evaluation following ISO 8586:2023 [80] and ISO 8587:2006 [81] standards. Using a 5-point scale, they assessed aroma intensity, aroma desirability, juiciness, tenderness, taste intensity and taste desirability, as outlined by Stanisławczyk et al. [82].Sensory evaluations were performed by a permanent laboratory team consisting of 12 panelists, each experienced in evaluating the sensory attributes of meat and meat products. Sensory evaluation was conducted during the day at room temperature in individual white-light booths. Samples were selected for sensory evaluation in random order. The evaluation panel had an equal 50% female-to-50 male distribution, and the panelists ranged in age from 35 to 55 years old. Before testing each sample, the evaluators took a 30 s break and rinsed their mouths with mineral water. The evaluation was conducted in 14 sessions, each with 12 samples.

### 3.4. Statistical Analysis

All observations composing the experiment (14 half-carcasses × 2 age groups × 6 treatments = 168 meat samples) were included in the statistical analysis. All analyses were conducted in triplicate, and the obtained results underwent statistical analysis subsequent to grouping. Statistical analysis was conducted using the Statistica 13.3PL package from TIBCO Software Inc. (Palo Alto, CA, USA). Utilizing the GLM procedure in Statistica (STATISTICA v. 10; StatSoft, Krakow, Poland), a two-factor analysis of variance (ANOVA) was applied to examine the chemical composition, selected physicochemical properties, color parameters and the level of pigments, texture, and sensory evaluations of the horse meat. When significance was observed (*p* < 0.05), means were compared using the post hoc Tukey honestly significant difference test. Table 1, Table 2 and Table 3 present mean values and standard error of individual qualitative features of meat.

## 4. Conclusions

Using high-pressure techniques alone (400 MPa for 15 min), or in combination with other heat treatment, did not improve all quality parameters of horse meat. Various treatment variants resulted in a lighter color of horse meat (L*) in both age groups of horses. The HHP technique led to the lowest scores for selected sensory quality parameters, while the combination of TC and S-V resulted in a deterioration of texture parameters, an increase in the TBARS index, weight loss, and an increase in MMb levels. The intensification of unfavorable trends resulting from the use of the aforementioned techniques was more noticeable in meat from older horses. Therefore, especially in the case of meat obtained from this age group, to limit the adverse impact of the techniques used on the aforementioned quality characteristics of horse meat, it is recommended to conduct further studies that modify the pressure and duration of high pressure, also in combination with other treatments or factors. Further research is advisable and justified to determine the temperature range and duration of heat treatment that will demonstrate improvement in the analyzed properties of horse meat. It is also advisable to conduct studies using proteolytic enzymes (papain, bromelain) or functional additives (e.g., phosphates in the meat industry). To minimize oxidation, the use of natural antioxidants may be a solution. Further studies would also be important to demonstrate potential changes in the protein structure of horse meat and how different processing variants affect the content of compounds responsible for the flavor and aroma of this raw material.

## Figures and Tables

**Table 1:** Chemical composition of horse meat as influenced by different processing methods (means ± SE).

Specification	Age	CS	HHP	TC	S-V	HHP + TC	HHP + S-V	ANOVA	*p*-Value
Protein (%)	Y O	20.20 ^a^ ± 0.13 20.50 ^a^ ± 0.14	20.15 ^a^ ± 0.14 20.78 ^a^ ± 0.77	20.10 ^a^ ± 0.10 20.30 ^a^ ± 0.11	19.90 ^a^ ± 0.09 20.90 ^a^ ± 0.12	20.10 ^a,x^ ± 0.07 21.45 ^a,y^ ± 0.01	20.20 ^a^ ± 0.01 20.40 ^a^ ± 0.14	A × T	0.0005
Fat (%)	Y O	3.98 ^a,x^ ± 0.47 7.15 ^a,y^ ± 0.84	3.05 ^a,b,x^ ± 0.27 8.00 ^a,b,y^ ± 0.77	5.63 ^c,x^ ± 0.24 8.95 ^b,y^ ± 0.56	4.10 ^a,b,x^ ± 0.21 7.15 ^a,y^ ± 0.09	5.20 ^b,c,x^ ± 0.28 9.70 ^b,y^ ± 0.01	3.80 ^a,x^ ± 0.01 6.90 ^a,y^ ± 0.41	A × T A T	0.0001 0.0001 0.0001
Water (%)	Y O	73.80 ^a,x^ ± 0.62 71.25 ^a,y^ ± 0.27	75.25 ^a,b,x^ ± 0.07 69.35 ^a,b,y^ ± 0.77	73.43 ^a,x^ ± 0.12 70.15 ^b,y^ ± 0.34	74.90 ^a,x^ ± 0.14 70.10 ^b,y^ ± 0.50	73.00 ^b,x^ ± 0.21 68.25 ^b,y^ ± 0.01	74.50 ^a,x^ ± 0.01 72.05 ^a,y^ ± 0.76	A × T A T	0.0001 0.0001 0.0001

Explanatory notes: CS—control samples, HHP—high hydrostatic pressure (400 MPa—15 min), TC—traditional cooking in vacuum-sealed foil bag in water at 100 °C for 1.5 h; S-V—sous-vide method in vacuum-sealed foil bag in water at 85 °C for 4 h; HHP + TC—high hydrostatic pressure and traditional cooking in vacuum-sealed foil bag in water at 100 °C for 1.5 h, HPP + S-V—high hydrostatic pressure and sous-vide method in vacuum-sealed foil bag in water at 85 °C for 4 h; ^a,b,c^—values indicated by different letters in the rows show statistically significant differences across the types of thermal treatments—*p* < 0.05; ^x,y^—values indicated by different letters in the columns show statistically significant differences across the age of animals—*p* < 0.05; ANOVA, two-way ANOVA analysis among the type of thermal treatments T and age of animal, A; Y—younger horses; O—older horses.

**Table 2:** Physicochemical characteristics of horse meat based on the type of processing (means ± SE).

Specification	Age	CS	HHP	TC	S-V	HHP + TC	HHP + S-V	ANOVA	*p*-Value
Forced drip [cm^2^]	Y O	5.82 ^x^ ± 0.08 2.94 ^y^ ± 1.11	5.48 ^x^ ± 0.40 3.97 ^y^ ± 0.16					A	0.0001
Thermal drip [%]	Y O	27.92 ^x^ ± 0.58 21.91 ^y^ ± 0.74	23.04 ^x^ ± 0.21 17.32 ^y^ ± 0.27					A	0.0001
TBARS [mg MDA/kg]	Y O	1.13 ^a,x^ ± 0.01 1.88 ^a,y^ ± 0.01	2.17 ^b^ ± 0.01 2.44 ^b^ ± 0.01	1.26 ^a,x^ ± 0.01 1.93 ^a,y^ ± 0.01	1.11 ^a^ ± 0.01 1.34 ^a^ ± 0.01	2.21 ^b^ ± 0.01 2.57 ^b^ ± 0.01	2.37 ^b^ ± 0.01 2.67 ^b^ ± 0.01	A × T A	0.0001 0.0001
Water activity	Y O	0.981 ± 0.01 0.983 ± 0.01	0.985 ± 0.01 0.984 ± 0.01	0.987 ± 0.01 0.984 ± 0.01	0.983 ± 0.01 0.980 ± 0.01	0.980 ± 0.01 0.981 ± 0.01	0.981 ± 0.01 0.987 ± 0.01		
Oxidation– reduction potential [mV]	Y O	368.00 ^a,x^ ± 0.01 390.00 ^a,y^ ± 0.01	389.40 ^b,x^ ± 0.01 400.30 ^a,b,y^ ± 0.01	359.89 ^a,x^ ± 0.02 385.34 ^a,y^ ± 0.01	361.67 ^a,x^ ± 0.01 381.20 ^a,y^ ± 0.01	403.00 ^c^ ± 0.01 408.00 ^a,b^ ± 0.01	398.00 ^b,c,x^ ± 0.01 410.00 ^b,y^ ± 0.01	A × T A	0.0001 0.0001
Weight loss [%]	Y O		42.00 ^a^ ± 0.01 39.28 ^a^ ± 0.01	48.56 ^b^ ± 0.01 45.92 ^b^ ± 0.02	40.76 ^a^ ± 0.01 38.59 ^a^ ± 0.01	46.46 ^c^ ± 0.01 43.06 ^c^ ± 0.01	45.82 ^c^ ± 0.01 42.91 ^c^ ± 0.01	T	0.0001

Explanatory notes: CS—control samples, HHP—high hydrostatic pressure (400 MPa—15 min), TC—traditional cooking in vacuum-sealed foil bag in water at 100 °C for 1.5 h; S-V—sous-vide method in vacuum-sealed foil bag in water at 85 °C for 4 h; HHP + TC—high hydrostatic pressure and traditional cooking in vacuum-sealed foil bag in water at 100 °C for 1.5 h, HPP + S-V—high hydrostatic pressure and sous-vide method in vacuum-sealed foil bag in water at 85 °C for 4 h; ^a,b,c^—values indicated by different letters in the rows show statistically significant differences across the types of thermal treatments—*p* < 0.05; ^x,y^—values indicated by different letters in the columns show statistically significant differences across the age of animals—*p* < 0.05; ANOVA, two-way ANOVA analysis among the type of thermal treatments T and age of animal, A; Y—younger horses; O—older horses.

**Table 3:** Texture parameters of horse meat based on the type of processing (means ± SE).

Specification	Age	CS	HPP	TC	S-V	HPP + TC	HPP + S-V	ANOVA	*p*-Value
Shear force [N/cm^2^]	Y O	40.18 ^a^ ± 1.17 60.49 ^a^ ± 3.60	88.75 ^b^ ± 8.16 98.29 ^b^ ± 7.86	129.34 ^d^ ± 5.11 146.33 ^d^ ± 3.90	126.78 ^d^ ± 4.12 136.34 ^d^ ± 5.11	142.24 ^c,x^ ± 2.77 181.42 ^c,y^ ± 4.15	141.91 ^c^ ± 6.48 157.93 ^c^ ± 8.90	A × T A T	0.0005 0.0001 0.0001
Hardness 1 [N]	Y O	192.07 ^a^ ± 11.18 210.73 ^a^ ± 10.69	239.82 ^b^ ± 12.73 246.59 ^b^ ± 12.23	243.67 ^b^ ± 4.56 260.67 ^b^ ± 3.22	233.34 ^b^ ± 8.45 250.87 ^b^ ± 9.98	289.74 ^c^ ± 11.43 299.54 ^c^ ± 12.38	271.45 ^c^ ± 11.33 288.92 ^c^ ± 11.57	T	0.0001
Hardness 2 [N]	Y O	116.76 ^a^ ± 10.21 129.78 ^a^ ± 11.72	143.19 ^b^ ± 11.06 172.37 ^b^ ± 11.07	140.33 ^b^ ± 4.11 164.32 ^b^ ± 4.01	145.67 ^b^ ± 7.43 158.32 ^b^ ± 9.12	161.55 ^c^ ± 15.19 181.62 ^c^ ± 13.10	160.98 ^c^ ± 19.01 174.47 ^c^ ± 13.20	T	0.0019
Stiffness 5 [N]	Y O	14.18 ^a^ ± 1.37 19.37 ^a^ ± 1.65	16.52 ^b^ ± 2.51 25.60 ^b^ ± 1.86	16.14 ^b^ ± 1.01 22.66 ^b^ ± 1.45	16.45 ^b^ ± 1.87 22.23 ^b^ ± 2.11	18.89 ^c^ ± 2.59 28.11 ^c^ ± 2.57	20.35 ^c^ ± 1.66 28.69 ^c^ ± 1.86	T	
Stiffness 8 [N]	Y O	30.28 ^a^ ± 6.68 49.66 ^a^ ± 5.87	35.55 ^b^ ± 4.94 56.26 ^b^ ± 5.70	37.67 ^b^ ± 1.12 51.33 ^b^ ± 4.23	38.45 ^b^ ± 2.05 56.34 ^b^ ± 1.45	45.78 ^c^ ± 4.42 68.93 ^c^ ± 4.02	48.21 ^c^ ± 5.09 70.78 ^c^ ± 20.41	T	0.0001
Adhesiveness (mJ)	Y O	1.93 ± 0.20 2.13 ± 0.92	2.43 ± 0.25 2.73 ± 0.01	2.45 ± 0.13 2.78 ± 0.14	1.87 ± 0.13 2.03 ± 0.11	2.10 ± 0.01 2.13 ± 0.78	2.66 ± 0.72 2.30 ± 0.26		
Cohesiveness	Y O	0.20 ± 0.03 0.20 ± 0.07	0.22 ± 0.09 0.24 ± 0.07	0.23 ± 0.04 0.56 ± 0.03	0.30 ± 0.01 0.35 ± 0.01	0.27 ± 0.03 0.28 ± 0.06	0.29 ± 0.05 0.31 ± 0.05		
Springiness [mm]	Y O	4.83 ± 0.26 5.52 ± 0.73	4.92 ± 0.52 5.18 ± 0.23	5.19 ± 0.17 5.86 ± 0.21	4.98 ± 0.30 6.55 ± 0.32	5.22 ± 0.52 5.29 ± 0.90	5.49 ± 0.51 5.53 ± 0.27		
Resilience	Y O	0.08 ± 0.02 0.09 ± 0.01	0.09 ± 0.02 0.15 ± 0.04	0.11 ± 0.01 0.23 ± 0.02	0.19 ± 0.01 0.32 ± 0.02	0.11 ± 0.02 0.11 ± 0.01	0.15 ± 0.02 0.13 ± 0.01		
Chewiness [mJ]	Y O	185.53 ^a^ ± 9.60 219.15 ^a^ ± 10.08	259.58 ^b^ ± 11.71 306.56 ^b^ ± 6.18	284.66 ^b^ ± 8.69 324.67 ^b^ ± 9.21	295.45 ^b^ ± 9.98 325.54 ^b^ ± 7.35	408.35 ^c^ ± 9.06 443.67 ^c^ ± 11.79	432.13 ^c^ ± 13.82 495.29 ^c^ ± 10.88	T	

Explanatory notes: CS—control samples, HHP—high hydrostatic pressure (400 MPa—15 min), TC—traditional cooking in vacuum-sealed foil bag in water at 100 °C for 1.5 h; S-V—sous-vide method in vacuum-sealed foil bag in water at 85 °C for 4 h; HHP + TC—high hydrostatic pressure and traditional cooking in vacuum-sealed foil bag in water at 100 °C for 1.5 h, HPP + S-V—high hydrostatic pressure and sous-vide method in vacuum-sealed foil bag in water at 85 °C for 4 h; ^a,b,c,d^—values indicated by different letters in the rows show statistically significant differences across the types of thermal treatments—*p* < 0.05; ^x,y^—values indicated by different letters in the columns show statistically significant differences across the age of animals—*p* < 0.05; ANOVA, two-way ANOVA analysis among the type of thermal treatments T and age of animal, A; Y—younger horses; O—older horses.

## Data Availability

The original contributions presented in the study are included in the article; further inquiries can be directed to the corresponding authors.

## Supplementary Materials

The following supporting information can be downloaded at: https://www.mdpi.com/article/10.3390/molecules30183749/s1, Table S1: Color parameters and the level of pigments in horse meat depending on the type of processing; Table S2: Sensory characteristics of horse meat based on the type of processing.
