# Supplementary material for: The Influence of Horse Age, High-Pressure Technique and Various Heat Treatment Methods on the Quality of Horse Meat
Source: Molecules. 2025 Sep 15;30(18):3749. doi: 10.3390/molecules30183749 (PMC12472574; doi:10.3390/molecules30183749)
Supplement: Supplementary file 1 [file molecules-30-03749-s001.zip › molecules-3827070-supplementary.pdf]

Table S1. Color parameters and the level of pigments in horse meat depending on the type of processing (means±SE).

Specification	Age	CS	HHP	TC	S-V	HHP + TC	HHP + S-V	ANOVA	*p*- Value
L*	Y	37.47^{a,x}±0.30	55.84^{b,x}±0.37	58.67^{b,x}±0.37	63.34^{c,x}±0.45	57.30^{b}±0.65	69.48^{d,x}±0.67	A×T	0.0173
	O	30.86^{a,y}±0.65	50.63^{b,y}±0.94	47.98^{b,y}±0.39	52.76^{c,y}±0.29	54.50^{c}±0.38	56.06^{d,y}±0.69	A	0.0024
								T	0.0001
a*	Y	19.01^{a}±0.57	13.11^{b,x}±0.92	11.45^{b,x}±0.51	15.23^{c,x}±0.22	10.67^{b,x}±0.11	17.21^{a,x}±0.98	A×T	0.0001
	O	19.42^{a}±0.15	16.75^{b,y}±0.23	14.54^{b,y}±0.34	17.55^{c,y}±0.47	13.65^{b,y}±0.25	19.43^{a,y}±0.94	A	0.0002
								T	0.0001
b*	Y	6.02^{a}±0.87	12.23^{b}±0.27	11.67^{b}±0.12	12.11^{b}±0.13	13.69^{b}±0.62	13.36^{b}±0.53	A×T	0.0019
	O	7.37^{a}±0.02	14.12^{b}±0.84	13.56^{b}±0.11	14.09^{b}±0.29	14.22^{b}±0.27	14.85^{b}±0.71	T	0.0001
Mb [%]	Y	27.99^{a}±0.28	12.71^{b,x}±0.49	29.45^{c,x}±0.56	31.05^{c,x}±0.36	17.20^{d,x}±0.25	25.78^{a,x}±0.78	A×T	0.0001
	O	29.15^{a}±0.36	33.22^{b,y}±0.04	55.78^{c,y}±0.89	57.34^{c,y}±0.29	42.38^{d,y}±0.21	54.23^{c,y}±0.21	A	0.0004
								T	0.0001
MMb [%]	Y	21.88^{a}±0.90	28.03^{a,b,x}±0.29	21.45^{a,x}±0.15	19.87^{a,x}±0.22	32.54^{c,x}±0.11	29.90^{d,x}±0.54	A×T	0.0001
	O	26.50^{a}±0.22	35.99^{b,y}±0.33	29.67^{a,y}±0.34	27.78^{a,y}±0.19	52.26^{c,y}±0.13	53.26^{c,y}±0.85	A	0.0381
								T	0.0001
$Mb \bullet O_2$ [%]	Y	44.33^{a}±0.10	38.74^{a,x}±0.85	22.98^{b}±1.05	17.67^{c}±0.34	25.07^{b}±0.89	15.86^{c}±0.78	A×T	0.0030
	O	50.12^{a}±0.32	49.28^{a,y}±0.28	27.56^{b}±0.98	22.45^{c}±0.78	30.56^{b}±0.31	20.95^{c}±0.71	T	0.0001
OZB [mg/g]	Y	434.54^{a,x}±4.32	180.16^{b,x}±2.75	426.98^{d,x}±6.21	324.40^{c,x}±5.45	288.29^{c,x}±2.33	177.65^{b,x}±2.28	A×T	0.0001
	O	702.23^{a,y}±6.12	277.08^{b,y}±1.66	524.78^{d,y}±3.78	529.45^{d,y}±6.48	354.36^{c,y}±1.49	398.28^{c,y}±2.38	A	0.0001
								T	0.0001

Explanatory notes: CS—control samples, HHP—high hydrostatic pressure (400 MPa—15 min), TC—traditional cooking in vacuum-sealed foil bag in water at 100 °C for 1.5 h; S-V—sous-vide method in vacuum-sealed foil bag in water at 85 °C for 4 h; HHP + TC—high hydrostatic pressure and traditional cooking in vacuum-sealed foil bag in water at 100 °C for 1.5 h, HPP + S-V—high hydrostatic pressure and sous-vide method in vacuum-sealed foil bag in water at 85 °C for 4 h; ^{a,b,c,d}—values indicated by different letters in the rows show statistically significant differences across the types of thermal treatments—$p < 0.05$; ^{x,y}—values indicated by different letters in the columns show statistically significant differences across the age of animals—$p < 0.05$; ANOVA, two-way ANOVA analysis among the type of thermal treatments T and age of animal, A; Y—younger horses; O—older horses.

Table S2. Sensory characteristics of horse meat based on the type of processing (means±SE).

Specification	Age	CS	HHP	TC	S-V	HHP + TC	HHP + S-V	ANOVA	*p-Value*
Aroma: intensity	Y	4.25±0.35	3.50±0.57	4.50±0.22	4.00±0.99	4.00±0.57	3.25±0.32		
	O	4.75±0.04	4.50±0.86	4.17±0.34	3.85±0.67	4.00±1.52	4.25±0.28		
Aroma: desirability	Y	4.25±0.76	3.00±0.57	4.33±0.11	4.12±0.12	4.25±0.28	3.00±0.50		
	O	4.25±0.50	3.75±0.50	3.50±0.23	3.50±0.09	3.75±0.50	3.00±0.76		
Juiciness	Y	2.75^{a}±0.28	2.00^{a}±0.15	2.83^{a}±0.09	4.25^{b}±0.50	3.75^{b}±0.04	4.00^{b}±0.15	T	0.0176
	O	2.50^{a}±0.57	1.00^{b}±0.01	1.85^{a}±0.09	4.00^{d}±0.75	3.00^{c}±0.86	2.75^{a,c}±0.25		
Tenderness	Y	2.75^{a}±0.86	1.00^{b}±0.57	3.00^{a}±0.32	4.00^{c}±0.51	3.50^{a,c}±0.57	1.25^{b}±0.01	T	0.0014
	O	1.60^{a}±0.86	1.50^{a}±0.57	1.85^{a}±0.11	4.25^{b}±0.23	2.50^{a}±0.28	1.75^{a}±0.57		
Taste: intensity	Y	3.50±0.50	3.25±0.76	4.17±0.06	4.50±0.10	4.00±0.57	4.50±0.01		
	O	4.50±0.28	4.00±1.15	4.00±0.22	4.50±0.12	3.75±0.86	4.50±0.28		
Taste: desirability	Y	3.25^{a}±0.76	2.00^{b}±0.01	3.33^{a}±0.21	4.00^{a}±0.20	3.75^{a}±0.28	2.50^{b}±0.50	T	0.0038
	O	3.50^{a}±0.86	1.75^{b}±0.50	3.00^{a}±0.34	4.25^{c}±0.21	3.50^{a}±1.00	2.50^{b}±0,57		

Explanatory notes: CS—control samples, HHP—high hydrostatic pressure (400 MPa—15 min), TC—traditional cooking in vacuum-sealed foil bag in water at 100 °C for 1.5 h; S-V—sous-vide method in vacuum-sealed foil bag in water at 85 °C for 4 h; HHP + TC—high hydrostatic pressure and traditional cooking in vacuum-sealed foil bag in water at 100 °C for 1.5 h, HPP + S-V—high hydrostatic pressure and sous-vide method in vacuum-sealed foil bag in water at 85 °C for 4 h; ^{a,b,c}—values indicated by different letters in the rows show statistically significant differences across the types of thermal treatments—$p< 0.05$; ^{x,y}—values indicated by different letters in the columns show statistically significant differences across the age of animals—$p< 0.05$; ANOVA, two-way ANOVA analysis among the type of thermal treatments T and age of animal, A; Y—younger horses; O—older horses.
